# Role of Intermediate dose escalation of radiotherapy in the survival of unresectable stage III non-small cell lung cancer patients in the era of immunotherapy

**DOI:** 10.3389/fonc.2025.1652522

**Published:** 2026-01-28

**Authors:** Saber A. Amin, Chi Lin, Apar K. Ganti, Weining Ken Zhen, Chi Zhang

**Affiliations:** 1 Department of Radiation Oncology, Fred & Pamela Buffett Cancer Center, University of Nebraska Medical Center, Omaha, NE, United States; 2 Division of Oncology-Hematology, University of Nebraska Medical Center, Fred and Pamela Buffett Cancer Center, Omaha, NE, United States; 3 Department of Radiation Oncology, Mayo Clinic Arizona, Phoenix, AZ, United States

**Keywords:** chemoradiation, chemotherapy, immunotherapy, national cancer database, non-small cell lung cancer, RT dose escalation

## Abstract

**Background:**

The role of dose escalation of radiotherapy (RT) in unresectable stage III NSCLC followed by immunotherapy is unclear. The objective of the current study is to investigate if intermediate dose escalation (IDE) is beneficial in stage III NSCLC patients who receive definitive concurrent chemoradiation (dcCRT) followed by immunotherapy.

**Materials and methods:**

The study used data from the National Cancer database. Multivariable Cox regression analysis was used to assess the all-cause mortality of patients who received standard RT dose (SD) (60 Gy ± 10%) and IDE (64–74 Gy).

**Results:**

47,315 patients were diagnosed in the era before immunotherapy and received dcCRT only, while 4,947 patients were treated with dcCRT and immunotherapy. In the cohort with dcCRT only, patients who received SD had statistically significant worse mortality but clinically minimal difference compared to patients with IDE (HR: 1.09, 95% CI: 1.07-1.12; p<0.0001). In the era of immunotherapy, SD was still associated with worse mortality compared to IDE (HR: 1.17, 95% CI: 1.03-1.33; p=0.02). However, the survival benefit associated with IDE was only restricted to patients who started immunotherapy within six weeks after RT completion (HR: 1.26, 95% CI: 1.05-1.6; p=0.01). There was no difference in mortality between SD and IDE among patients who started immunotherapy between 7–10 weeks (HR: 1.13, 95% CI: 0.88-1.45; p=0.35) or >10 weeks after RT completion (HR: 0.74, 95% CI: 0.51-1.07; p=0.11).

**Conclusion:**

IDE of RT is not needed for patients diagnosed with stage III unresectable NSCLC who receive immunotherapy > six weeks after dcCRT.

## Introduction

Lung cancer is the leading cause of cancer death in the United States (1). More Than 85% of the patients with lung cancer have non-small cell lung cancer (NSCLC) (2). One-third of NSCLC patients have stage III disease, among whom 70% are unresectable (3). The five-year survival rate of stage III NSCLC patients is between 15-30% (4).

Historically unresectable stage III NSCLC patients with good performance status were treated with platinum-based doublet chemotherapy given concurrently with radiotherapy, *i.e.*, definitive concurrent chemoradiation (dcCRT) (5). However, despite providing a locoregional control rate of 60-70%, progression-free survival (PFS) and overall survival (OS) remains poor (6). Before durvalumab approval, radiation dose escalation was studied to improve local control and OS of patients with unresectable stage III NSCLC. The current standard radiation dose of 60 Gy was established by the Radiation Therapy Oncology Group (RTOG) 7301 trial and has remained unchanged since the 1970s (7). However, since then, multiple attempts have been made to determine the optimal RT dose for the management of stage III NSCLC as ideally, increasing the RT dose should improve local and regional control and OS (4, 8–15). Prospective trials demonstrated safety for dose escalation to >80Gy if standard lung dose constraints are followed with definitive RT only and an intermediate dose-escalation from 60 Gy to 66Gy with concurrent chemotherapy is safe but showing no significant difference in overall survival (16, 17). A large, randomized phase III, multi-institutional RTOG 0617 trial was developed prior to the immunotherapy era, which tested the role of RT dose escalation (74 Gy) given concurrently with chemotherapy and or cetuximab in compared with standard chemoradiotherapy (60 Gy) plus or minus cetuximab (9). RTOG 0617 trial failed to show any survival improvement with 74 Gy or with cetuximab added to the standard regimen (18, 19). Contrary to expectation, 74 Gy was associated with worse OS than 60 Gy (9). Due to the unexpected results of the trial, various researchers have tried to explain these findings (8, 20, 21). Certain factors are believed to have contributed to the worse OS of 74 Gy RT dose, such as higher treatment-related mortality, difficulty completing concurrent chemotherapy, and the higher likelihood of non-compliant RT planning in the higher RT dose compared to 60 Gy (9). Overall survival was still worse in the 74 Gy arm when some of these factors were eliminated (8). One particular concern of dose escalation is increased immunosuppression due to RT-induced immune cell depletion with a higher dose of RT (22, 23). The conclusion was that 60Gy should remain the standard RT dose for unresectable stage III NSCLC and dose higher than 74Gy should be avoided (9). However, the RTOG 0617 trial compared two different RT doses by comparing 74 Gy with 60 Gy and not including patients who received doses between 60 Gy and 74 Gy (9). Whether an intermediate dose escalation (IDE) (between 64–74 Gy) is associated with any clinical benefit remains unclear for the patients treated with dcCRT only.

The role of IDE is now an even more pertinent question in clinical practice because dcCRT followed by durvalumab is now the standard of care in unresectable stage III NSCLC patients after the findings from the landmark PACIFIC trial randomizing patients after the end of the dcCRT remaining in good performance status to durvalumab *versus* placebo (24, 25). Adjuvant durvalumab, in addition to dcCRT, was associated with improved PFS and OS (24). Although the PACIFIC trial showed significantly improved survival, the intrathoracic failure rate was high (36%), which could be attributed to the lower RT dose as most patients received an RT dose between 54–66 Gy (24). Subsequent PACIFIC-R, an observational/non-interventional, retrospective study showed that about half of the patients with unresectable stage III NSCLC treated on PACIFIC regimen in real world had received dose of RT > 60Gy although no details of the dose are available in the report or analyzed for survival (26). This study does suggest that whether there is any benefit for intermediate dose escalation to < 74Gy remains a question to be answered in the community. A couple of small retrospective studies have reported that the combination of higher-dose radiation (< 74Gy) with chemotherapy followed by durvalumab is safe and has similar toxicity, local control rate, and distant control rate to what has been reported in the PACIFIC trial (27, 28). One study with 39 stage III NSCLC patients who received RT dose > 66 Gy with concurrent chemotherapy followed by durvalumab reported that this regimen is safe and might improve outcomes in selected stage III NSCLC patients. Thoracic failure was 21%, and 12-month OS was 79%. In contrast, in the PACIFIC trial, thoracic failure was 38%, and 12-month OS was 81% (27). Another study conducted in Europe included 78 patients and compared an RT dose of 73.5 Gy with 66 Gy followed by durvalumab and reported no difference in local control, regional control, and distant control, and recommended that RT dose escalation could safely be combined with durvalumab (28). It is noted that 73.5 Gy higher dose of RT was delivered with sequential chemotherapy while 66Gy was delivered concurrently with chemotherapy in this study. However, whether the safe intermediate escalated dose is beneficial in prolonging survival remains inconclusive due to the small size of these studies and lack of consistency in treatment regimen selection. No prospective study has investigated the role of dose escalation, particularly IDE, in the OS of unresectable stage III NSCLC in the era of immunotherapy after the approval of durvalumab.

The current study aims to utilize the National Cancer Database (NCDB) to investigate the association of IDE with the OS of unresectable stage III NSCLC patients before and particularly after the era of immunotherapy. We hypothesize that an intermediate RT dose escalation, *i.e.*, up to 74Gy, may not be needed with concurrent chemoradiation followed by immunotherapy for unresectable stage III NSCLC.

## Materials and methods

### Data source

Data were extracted from the NCDB, a nationwide oncology outcomes database for more than 1500 Commission on Cancer-accredited cancer programs in the United States and Puerto Rico. It is a multi-center hospital-based cancer registry, which collects >70% of cancer cases diagnosed in the U.S. annually from hospital cancer registries across the country. De-identified data were used; therefore, the study was determined to be exempt from Institutional Review Board (IRB) review.

### Study population

Patients aged 18 and older who received concurrent chemoradiation (within 30 days of each other) and were diagnosed with unresectable stage III NSCLC between 2004–2020 were included in this study. All patients received multiagent chemotherapy. We excluded patients who received surgery or with missing surgery, chemotherapy, radiation therapy, or immunotherapy information. Patients who only received chemotherapy or RT and patients with RT dose <57 Gy or >74 Gy were also excluded. We also excluded patients who started chemotherapy and RT non-concurrently, *i.e*., > 30 days from each other (29). We divided the study population into two cohorts. The cohort before the era of immunotherapy included patients diagnosed between 2004 and 2016, while the cohort after the era of immunotherapy included patients diagnosed with unresectable stage III NSCLC between 2017 and 2020. Patients who received immunotherapy between 2004–2015 were excluded from the cohort before the immunotherapy era. For the immunotherapy cohort, all patients received immunotherapy. Patients who started immunotherapy before the completion of RT or >180 days after RT completion or started chemotherapy after immunotherapy or immunotherapy >180 days after chemotherapy were excluded.

### Outcome and covariables

The primary endpoint/outcome of interest for this study was OS. OS but not PFS, local control or toxicity was the only available outcomes data provided in NCDB. The main covariable of interest was the dose of RT. Other covariables of interest included age at diagnosis (years), insurance status (yes or no), race and ethnicity, Charlson-Deyo Comorbidity Index (0, 1, ≥2), treatment facility type (academic/research or non-academic), year of diagnosis, neighborhood education level, T, N and group stages and median household income. Neighborhood education level in the NCDB is documented by matching the patient zip code recorded at the time of diagnosis with the American Community Survey 2016 and categorized into quartiles of adults ≥ 25 years who did not graduate from high school (≥ 17.6%, 10.9–17.5%, 6.3–10.8%, and <6.3%), which we combined into two groups (<10.9% as higher education level and ≥ 10.9% as the lower education level). Median household income in the NCDB is derived from matching the patient zip code recorded at the time of diagnosis with the American Community Survey 2016 and categorized into quartiles (<$40,227, $40,227–$50,353, $50,354–$63,332, and ≥ $63,333), which we combined into two groups (≥ $50,353 or <$50,353). The sequence of immunotherapy with RT was divided into three groups, immunotherapy within six weeks of RT completion, 7–10 weeks after RT completion, and > 10 weeks after RT completion. The sequence of immunotherapy with chemotherapy was divided into starting immunotherapy within ten weeks, between 11–14 weeks, and >14 weeks after starting chemotherapy. These timing windows between starting chemotherapy and immunotherapy correlate with the time intervals between end of RT to start of IO being within 4 weeks, 5–8 weeks, and > 8 weeks, respectively, expecting an average duration of chemotherapy at dcCRT step being 6 weeks.

### Statistical analysis

Descriptive characteristics by race and ethnicity were calculated as mean and standard deviation (SD) for continuous variables and frequency and percent for categorical variables. Odds ratios (OR) and 95% confidence intervals (CI) were estimated using multivariable logistic regression models to identify factors associated with receiving escalated RT dose (64–74 Gy).

We used the Kaplan-Meier survival curves to estimate the median survival time and computed the log-rank test to compare survival time across RT dose groups. Cox proportional hazard regression models were used to estimate hazard ratios (HR) and 95% CI for dose escalation in the era before immunotherapy (2004-2016) and the era of immunotherapy (2017-2020) for all-cause mortality in patients diagnosed with unresectable stage III NSCLC. Survival time was measured in months, beginning from the date of diagnosis to the date of death or last follow-up. Patients alive at the last follow-up or lost to follow-up were censored. All statistical analyses were performed with SAS version 9.4 (SAS Institute Carey, NC).

## Results

The sample size for the cohort before the era of immunotherapy included (47,315) patients, while the immunotherapy cohort included (4749) patients. These cohorts are generated based on selection criteria for patients who received concurrent dcCRT followed by immunotherapy, after excluding all other stages except stage III, excluding those who received surgery, and excluding those who received a dose <56 Gy and more than 80Gy. The demographic data of both cohorts are reported in **Table 1A**. Most of the patients were male, white, had squamous cell carcinoma, were treated at community hospitals, and had a comorbidity score of zero. Patients with an RT dose of 57–63 Gy were more likely to reside in higher-income neighborhoods and neighborhoods with higher education levels than patients who received an intermediately escalated RT dose (64–74 Gy). Patients who received RT dose 57–63 Gy were also more likely to be treated at academic hospitals than those who received RT dose 64–74 Gy. Patients with higher N stages or group stage of NSCLC were more likely to be treated with lower dose RT of 57–63 Gy. The absence of stage IIIC patients in pre-immunotherapy era (2004-2016) is consistent with the transition of AJCC 7^th^ edition of NSCLC staging system to 8^th^ edition in 2017.

**Table 1A:** Baseline characteristics of patients diagnosed with inoperable stage III NSCLC between 2004–2020 by RT dose for era before immunotherapy and era after its approval.

Variable		Before the era of immunotherapy (2004-2016)				The era of immunotherapy (2017-2020)			
		Dose 57-63 Gy *N*=23,324 (49.3%)	Dose 64-74 Gy *N*= 23,991 (50.7%)	Total *N*=47315	P	Dose 57-63 Gy *N*=3,210 (67.6%)	Dose 64-74 Gy *N*= 1,539 (32.4%)	Total *N*=4749	p
Age at diagnosis	Continuous (median and ranges)	65 (19-90)	65 (23-90)	67 (21-90)	0.001	67 (29-90)	66 (21-90)	67 (21-90)	0.22
Sex	Male	1,3308 (57.1)	14,168 (59.1)	27,476 (58.1)	0.001	1,744 (54.3)	890 (57.8)	2,634 (55.5)	0.02
	Female	10,016 (42.9)	9,823 (40.9)	19,839 (41.9)		1,466 (45.7)	649 (42.2)	2,115 (44.5)	
Race	White	19,679 (84.9)	20,263 (84.8)	39,942 (84.9)	0.001	2,690 (84.2)	1,286 (83.8)	3976 (84.1)	0.12
	Black	2,859 (12.3)	3091 (13.0)	5,950 (12.5)		387 (12.1)	206 (13.2)	593 (12.5)	
	Non-White non-Black	644 (2.8)	531 (2.2)	1,175 (2.5)		118 (3.7)	44 (3.0)	160 (3.4)	
Histology	Adenocarcinoma	8,134 (34.9)	7,909 (33.0)	16,043 (33.9)	0.001	1,446 (45.0)	703 (45.7)	2,149 (45.2)	0.98
	Squamous cell carcinoma	9,950 (42.7)	10,477 (43.7)	20,427 (43.2)		1,638 (51.0)	777 (50.5)	2,415 (50.8)	
	Large cell carcinoma	614 (2.6)	718 (3.0)	1,332 (2.8)		31 (1.0)	14 (0.9)	45 (1.0)	
	Undifferentiated	4,626 (19.8)	4,887 (20.3)	9,513 (20.1)		95 (3.0)	45 (2.9)	140 (3.0)	
Charlson/Deyo comorbidity score	0	14,402 (61.7)	14,847 (61.9)	29,249 (61.8)	0.01	1,841 (57.4)	867 (56.3)	2,708 (57.0)	0.14
	1	6,122 (26.3)	6,455 (26.9)	12,577 (26.6)		823 (25.6)	375 (24.4)	1,198 (25.2)	
	≥2	2,800 (12.0)	2,689 (11.2)	5,489(11.6)		546 (17.0)	297 (19.3)	843 (17.8)	
Neighborhood education level	≥10.9% NHD	10,448 (50.1)	11,770 (54.6)	22,218 (52.4)	0.001	1,333(49.3)	746 (58.3)	2,089 (52.2)	0.001
	<10.9% NHD	10,416 (49.9)	9,768 (45.4)	20,184 (47.6)		1,372 (50.7)	541 (41.7)	1,913 (47.8)	
Household income	<$50,353	9,691 (46.6)	11,316 (52.7)	21,007 (49.7)	0.001	1,251 (46.3)	706 (54.4)	1,957 (49.0)	0.001
	≥$50,353	11,114 (53.4)	10,170 (47.3)	21,284 (50.3)		1,449 (53.7)	591 (45.6)	2,040 (51.0)	
Treatment facility type	Academic	6,423 (27.7)	5,893 (24.7)	12,315 (26.2)	0.001	895 (28.0)	364 (23.8)	1,259 (26.6)	0.003
	Community	16,778 (72.3)	17,953 (75.3)	34,731(73.8)		2,305 (72.0)	1,165 (76.2)	3,472 (73.4)	
T stage	T1	3255 (15.0)	3216 (14.5)	6,471 (14.8)	0.31	574 (18.1)	259 (17.0)	833 (17.7)	0.45
	T2	6848 (31.6)	7017 (31.6)	13,865 (31.6)		733 (23.1)	330 (21.7)	1063 (22.6)	
	T3	4826 (22.3)	4936 (22.2)	9,762 (22.3)		759 (23.9)	376 (24.7)	1135 (24.2)	
	T4	6724 (31.1)	7033 (31.7)	13,757 (31.4)		1109 (34.9)	557 (36.6)	1666 (35.5)	
N stage	N0	1692 (7.8)	2108 (9.5)	3,800 (8.7)	0.001	305 (9.6)	183 (12.0)	488 (10.4)	0.001
	N1	1281 (5.9)	1493 (6.7)	2,774 (6.3)		250 (7.9)	146 (9.6)	396 (8.4)	
	N2	13324 (61.5)	14421 (65.0)	27,745 (63.3)		1891 (59.6)	943 (62.0)	2834 (60.3)	
	N3	5356 (24.7)	4180 (18.8)	9,536 (21.7)		729 (23.0)	250 (16.4)	979 (20.8)	
Group stage	IIIA	12,022 (55.5)	13,220 (59.5)	25,242 (57.6)	0.001	1548 (48.8)	822 (54.0)	2370 (50.5)	0.001
	IIIB	9,631 (44.5)	8,982 (40.5)	18,613 (42.4)		1335 (42.1)	606 (39.8)	1941 (41.3)	
	IIIC	Not defined in AJCC 7^th^ Edition				292 (9.2)	94 (6.2)	386 (8.2)	
Immunotherapy sequence with RT	Within 42 days of RT completion					1,857 (57.9)	902 (58.6)	2,759 (58.0)	0.2
	43-70 days of RT completion					922 (28.7)	409 (26.6)	1,331 (33.8)	
	>70 days of RT completion					431 (13.4)	228 (14.8)	659 (13.9)	
Immunotherapy sequence with chemo	Within 70 days after the start of chemotherapy					778 (24.2)	283 (18.4)	1,061 (13.6)	0.001
	71-98 days after the start of chemotherapy					1507 (47.0)	704 (35.9)	2,211 (50.0)	
	>98 days after the start of chemotherapy					925 (28.8)	552 (41.9)	1,477 (36.4)	

NHD, no high school degree.

P values: calculated for comparisons between cohorts of IDE (64-74Gy) and SD (57-63Gy) for each demographic category.

**Table 1B:** Percentage of patients by stage and immunotherapy sequence for the era after immunotherapy approval.

Immunotherapy sequence with RT	Group stage		
	IIIA	IIIB	IIIC
Within 42 days of RT completion	1361 (57.4%)	1128 (58.1%)	242 (62.7%)
43–70 days of RT completion	660 (27.9%)	556 (28.7%)	98 (25.4%)
>70 days of RT completion	349 (14.7%)	257 (13.2%)	46 (11.9%)

Group stage	Within 42 days of RT completion	43–70 days of RT completion	>70 days of RT completion
IIIA	1361 (49.8%)	660 (50.2%)	349 (53.5%)
IIIB	1128 (41.3%)	556 (42.3%)	257 (39.4%)
IIIC	242 (8.9%)	98 (7.5%)	46 (7.1%)

All percentages were calculated per the total number of each column.

Numerically higher percentages of patients with more advanced stage of disease started immunotherapy sooner after RT (**Table 1B**). For example, 8.9% of stage IIIC patients started within 6 weeks *vs.* 7.1% started > 10 weeks after RT, while 49.8% of stage IIIA patients started within 6 weeks *vs*. 53.5% started > 10 weeks after RT.

### Survival outcomes

#### Before the era of immunotherapy

**Figure 1:**
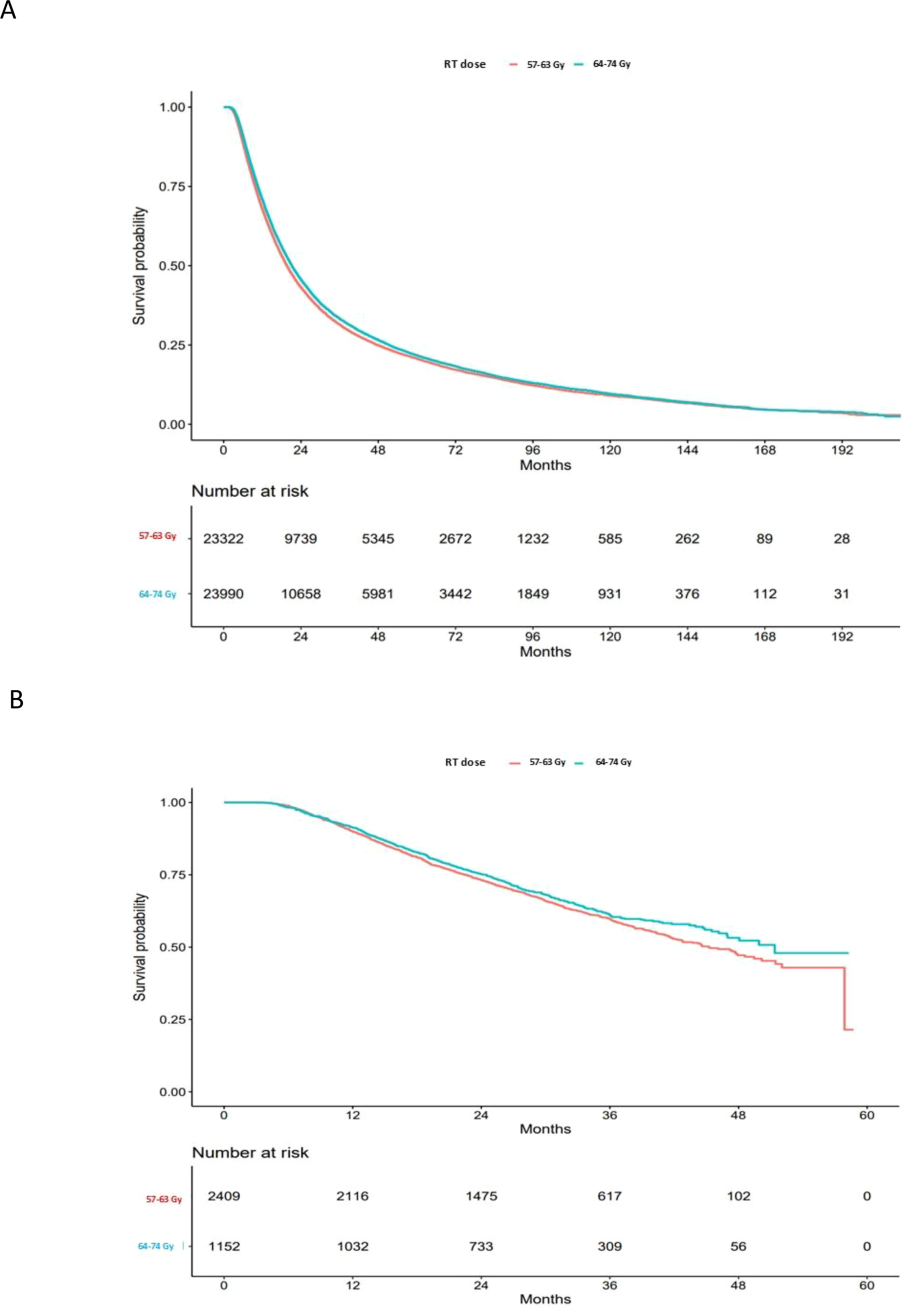
Overall survival of patients with intermediate dose escalation (64-74Gy) vs. standard RT dose (57-63Gy); in the era of pre-immunotherapy **(A)**, and for those received adjuvant immunotherapy after dcCRT **(B)**. Red curves: 57–63 Gy; blue curves: 64–74 Gy.

For the cohort of stage III NSCLC patients diagnosed between 2004 and 2016 who received definitive concurrent chemoradiation but no immunotherapy, the median follow-up time was 71 months, while the median survival time was 20.4 (95% CI: 20.2-20.7) months. The median survival time was 19.7 (95% CI: 19.3-20.0) months for patients who received an RT dose of 57–63 Gy and 21.2 (95% CI: 20.8-21.5) months for patients who received an RT dose of 64–74 Gy (p < 0.0001) (**Figure 1A**). In the multivariable Cox regression analysis, adjusted for age at diagnosis, sex, race, income, education, treatment facility type, comorbidity score, and histology, patients who received an RT dose of 57–63 Gy had worse all-cause mortality compared to patients with intermediate RT dose escalation (64–74 Gy) (HR: 1.09, 95% CI: 1.07-1.12; p<0.001) (**Table 2**).

**Table 2:** Multivariable analysis of RT dose for all-cause mortality among 47,315 patients diagnosed with inoperable stage III NSCLC between 2004-2016 (pre-immunotherapy era).

Variable		Multivariable analysis	P
		HR (95% CI)	
Age at diagnosis	Continuous (median and ranges)	1.01 (1.01-1.01)	0.001
RT dose	57–63 Gy	1.09 (1.07-1.12)	0.001
	64–74 Gy	Ref	
Sex	Male	Ref	0.001
	Female	0.83 (0.82-0.85)	
Race	White	Ref	
	Black	0.91 (0.88-0.94)	0.001
	Non-White non-Black	0.82 (0.76-0.88)	0.001
Histology	Adenocarcinoma	Ref	
	Squamous cell carcinoma	1.12 (1.09-1.15)	0.001
	Large cell carcinoma	1.13 (1.06-1.20)	0.001
	Other undifferentiated	1.11 (1.07-1.14)	0.001
Charlson/Deyo comorbidity score	0	Ref	
	1	1.08 (1.05-1.10)	0.001
	≥2	1.16 (1.12-1.20)	0.001
Neighborhood education level	≥10.9% NHD	Ref	0.03
	<10.9% NHD	0.97 (0.95-0.99)	
Household income	<$50,353	Ref	0.001
	≥$50,353	0.93 (0.91-0.96)	
Treatment facility type	Academic	0.91 (0.89-0.93)	0.001
	Community	Ref	

#### Era of immunotherapy

**Figure 2:**
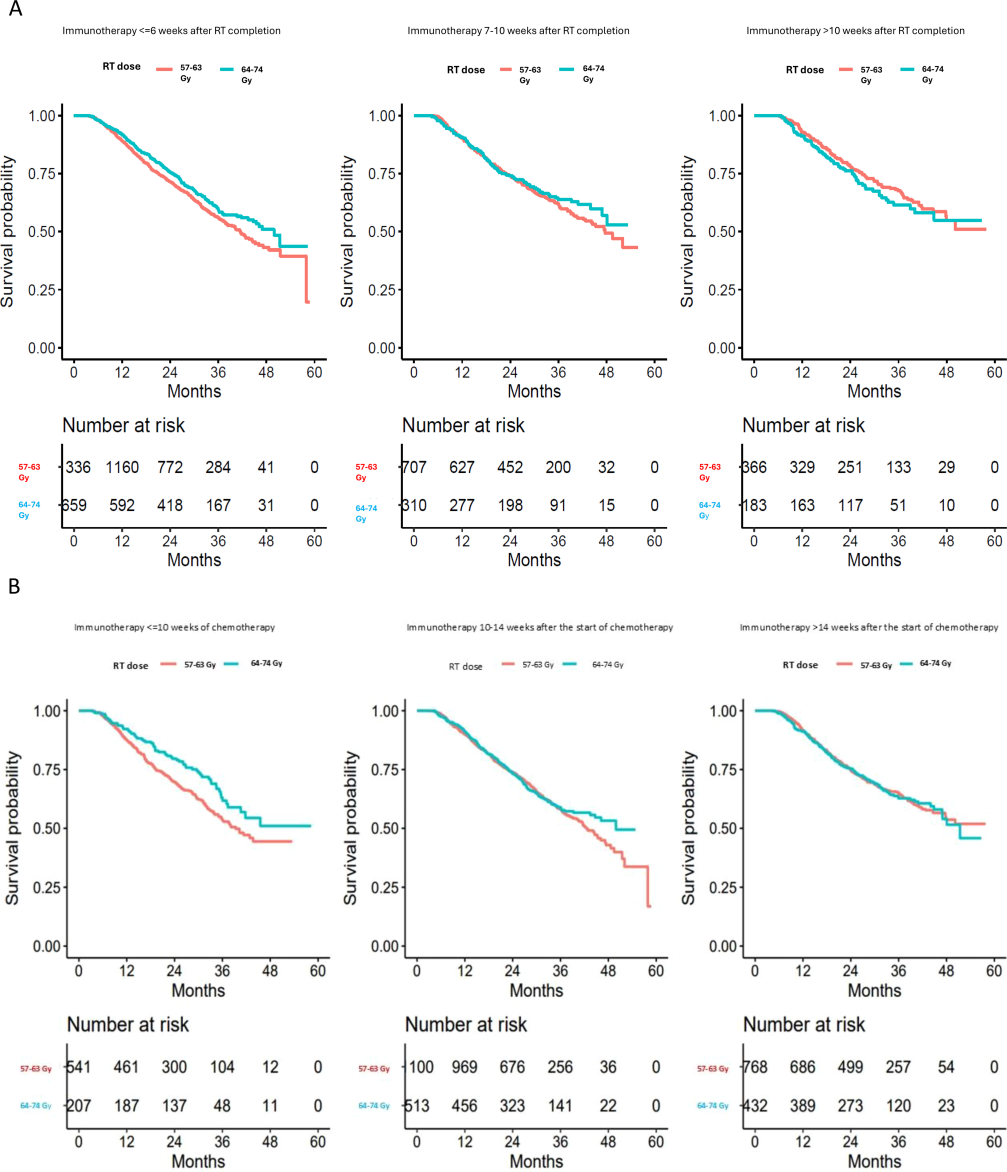
Overall survival of patients with intermediate dose escalation (64-74Gy) vs. standard RT dose (57-63Gy) stratified by starting time of immunotherapy after completion of RT **(A)**, or by starting time of immunotherapy after starting of chemotherapy **(B)**. Red curves: 57–63 Gy; blue curves: 64–74 Gy.

For the cohort of stage III NSCLC patients diagnosed between 2017 and 2020 who received definitive concurrent chemoradiation and adjuvant immunotherapy, the median follow-up time was 31 months, while the median survival time was 47.7 (95% CI: 44.7-51.4) months. The median survival time was 45.2 (95% CI: 42.2-49.5) months for patients who received an RT dose of 57–63 Gy and 51.4 (95% CI: 46.9-not reached) months for patients who received an RT dose 64–74 Gy (p = 0.0881) (**Figure 1B**). Among patients who started immunotherapy within six weeks of RT completion, the median OS was 41.5 (95% CI: 38.0-44.6) months for patients who received an RT dose of 57–63 Gy and 49.9 (95% CI: 43.4-not reached) months for patients who received an RT dose 64–74 Gy (p=0.03). Among patients who started immunotherapy > six weeks but ≤10 weeks after RT completion, the median OS was 47.7 (95% CI: 42.7-not reached) months for patients with 57–63 Gy and not reached (95% CI: 46.9-not reached) for patients with 64–74 Gy (p =0.38). Among patients who started immunotherapy > 10 weeks after RT completion, the median OS was not reached (95% CI: 47.8-not reached) for patients with 57–63 Gy and not reached (95% CI: 40.0-not reached) for patients with 64–74 Gy (p =0.40) (**Figure 2A**). Based on the above data, a trend of better OS was observed when immunotherapy started later than early in both the standard RT dose and IDE cohorts, respectively. Although the data suggested that patients with IDE had better OS than SD cohort if immunotherapy was started within 6 weeks after RT, the OS of these patients started with immunotherapy early even with dose escalation is not better than those with the standard dose of RT but waited for at least 6 weeks after RT to start immunotherapy (HR: 0.96, 95% CI: 0.80-1.14; p=0.66). Further analysis demonstrated that the survival benefits of IDE compared with SD (< 6 weeks) were most contributed by those who started immunotherapy within 2 weeks of completion of RT (HR: 2.19, 95% CI: 1.32-3.62, p=0.002 standard dose vs. IDE) with a 2-year survival rate of 65% (95% CI: 57%-73%) and 84% (95% CI: 76%-92%) for standard dose and IDE, respectively.

Among patients who started immunotherapy within 10 weeks of starting chemotherapy as a surrogate of about one month after completion of concurrent CRT, the median OS was 39.4 (95% CI: 35.1-not reached) months for patients who received an RT dose of 57–63 Gy and not reached (95% CI: 37.4-not reached) for patients who received an RT dose 64–74 Gy (p=0.01). Among patients who started immunotherapy >10 weeks but ≤ 14 weeks after the start of chemotherapy, the median OS was 42.7 (95% CI: 40.3-46.1) months for patients who received an RT dose of 57–63 Gy and 49.9 (95% CI: 44.7-not reached) months for patients who received an RT dose 64–74 Gy (p =0.43). Among patients who started immunotherapy >14 weeks after the start of chemotherapy, the median OS was not reached (95% CI: 47.7-not reached) months for patients who received an RT dose of 57–63 Gy and 51.4 (95% CI: 46.9-not reached) months for patients who received an RT dose 64–74 Gy (p=0.93) (**Figure 2B**).

In the multivariable (MVA) Cox regression analysis, RT dose 57–63 Gy was associated with worse all-cause mortality compared to intermediate RT dose escalation (64–74 Gy) (HR: 1.17, 95% CI: 1.03-1.34; p=0.02) (**Table 3A**). Other factors associated with improved all-cause mortality included female sex, African American, comorbidity score of >=2, adenocarcinoma histology, and immunotherapy>70 days after RT completion (**Table 3A**). As expected, group stages but not individual T and N stages trends toward significant association with all-cause mortality/OS. We also performed MVA Cox regression analysis for the OS of patients comparing SD (57–63 Gy) with IDE RT dose (64–74 Gy) for the cohorts of patients before and within immunotherapy era, respectively, stratified by T, N, and group stage (**Table 3B**). For the cohort in pre-immunotherapy era, SD is statistically significantly associated with increased risks of mortality in all T stages, higher N stages and group stages compared to IDE RT dose, with HR being consistently ~ 1.1. However, for the cohort who received immunotherapy, the benefit of IDE seems to diminish with none of the T or N stages showing significantly increased HR in SD of RT. It is even surprising to see the significantly lowered HR (0.86, 95% CI: 0.66-0.99, p=0.047) in SD RT group for stage IIIB patients compared to IDE RT dose (**Table 3B**).

**Table 3A:** Multivariable analysis of RT dose for all-cause mortality among 4,749 patients diagnosed with inoperable stage III NSCLC between 2017-2020 (immunotherapy era).

Variable		Multivariable analysis	P
		HR (95% CI)	
Age at diagnosis	Continuous (median and ranges)	1.01 (1.00-1.02)	0.04
RT dose	57-63 Gy	1.17 (1.03-1.33)	0.02
	64-74 Gy	Ref	
Immunotherapy sequence with RT	Within 42 days of RT completion	Ref	
	43-70 days of RT completion	0.91 (0.79, 1.04)	0.17
	>70 days of RT completion	0.82 (0.69-0.99)	0.02
Sex	Male	Ref	0.001
	Female	0.78 (0.69-0.89)	
Race	White	Ref	
	Black	0.61 (0.49-0.76)	0.001
	Non-White non-Black	0.91 (0.64-1.29)	0.60
Histology	Adenocarcinoma	Ref	
	Squamous cell carcinoma	1.44 (1.26-1.64)	0.001
	Large cell carcinoma	1.93 (1.11-3.37)	0.02
	Other undifferentiated	1.12 (0.79-1.60)	0.52
Charlson/Deyo comorbidity score	0	Ref	
	1	1.17 (1.01-1.34)	0.03
	≥2	1.21 (1.03-1.42)	0.02
Neighborhood education level	≥10.9% NHD	Ref	0.07
	<10.9% NHD	1.15 (0.99-1.32)	
Household income	<$50,353	Ref	0.04
	≥$50,353	0.86 (0.74-0.99)	
Treatment facility type	Academic	1.02 (0.89-1.17)	0.77
	Community	Ref	
T stage	T1	Reference	
	T2	1.21 (0.99- 1.49)	0.07
	T3	1.20 (0.92-1.57)	0.17
	T4	1.17 (0.87-1.57)	0.30
N stage	N0	Reference	
	N1	0.77 (0.57-1.04)	0.09
	N2	0.99 (0.72-1.35)	0.94
	N3	0.93 (0.56-1.53)	0.76
Group stage	IIIA	Reference	
	IIIB	1.28 (1.00-1.64)	0.05
	IIIC	1.44 (0.88-2.36)	0.15

**Table 3B:** MVA Cox regression for the OS of patients stratified by T, N, and group stage comparing standard dose (57–63 Gy) with IDE (64–74 Gy).

	Before the era of immunotherapy		Era of immunotherapy	
	HR (95% CI)	P	HR (95% CI)	P
T stage				
T1	1.09(1.02-1.15)	0.006	1.24 (0.86-1.80)	0.25
T2	1.09 (1.05-1.13)	0.001	1.08 (0.82-1.43)	0.60
T3	1.06 (1.01-1.11)	0.02	1.17 (0.89-1.54)	0.27
T4	1.11(1.07-1.15)	0.001	1.15 (0.91-1.44)	0.24
N stage				
N0	1.05 (0.97-1.13)	0.25	0.90 (0.60-1.35)	0.60
N1	1.07 (0.98-1.17)	0.13	1.17 (0.71-1.92)	0.55
N2	1.10 (1.07-1.13)	0.001	1.15 (0.97-1.36)	0.10
N3	1.07 (1.02-1.12)	0.008	1.31 (0.88-1.96)	0.18
Group stage				
IIIA	1.09 (1.06-1.12)	0.001	1.04 (0.86-1.25)	0.69
IIIB	1.09 (1.05-1.12)	0.001	0.81 (0.66-0.99)	0.047
IIIC	Not defined in AJCC 7^th^ Edition	n/a	1.37 (0.83-2.28)	0.22

Interestingly, when we stratified the cohort by the time between RT completion and immunotherapy initiation, we again found that the survival benefit associated with IDE was only restricted to patients who started immunotherapy within six weeks of RT completion (HR: 1.27, 95% CI: 1.08-1.51; p=0.01), concordant with the time interval used in the PACIFIC trial (**Table 4**). More importantly, there was no difference in all-cause mortality between RT dose of 57–63 Gy and IDE (64–74 Gy) among patients who started immunotherapy between 7–10 weeks after RT completion (HR: 1.13, 95% CI: 0.88-1.45; p=0.35) or patients who started immunotherapy >10 weeks after RT completion (HR: 0.74, 95% CI: 0.51-1.07; p=0.11) (**Table 4**).

**Table 4:** Hazard ratio and its 95% CI of RT dose for all-cause mortality stratified by immunotherapy time after RT completion.

Variable	Categories	HR 95% CI	P
Immunotherapy within 6 weeks of RT completion (42 days)			
RT dose	57–63 Gy	1.26 (1.05-1.6)	0.01
	64–74 Gy	Ref	
Immunotherapy between 7–10 weeks (43–70 days) after RT completion			
RT dose	57–63 Gy	1.13 (0.88-1.45)	0.35
	64–74 Gy	Ref	
Immunotherapy >10 weeks (70 days) after RT completion			
RT dose	57–63 Gy	0.74 (0.51-1.07)	0.11
	64–74 Gy	Ref	

The multivariable analysis was adjusted for age at diagnosis, gender, race, income, education, histology, comorbidity score, treatment facility type, T stage, N stage, and group stage.

When the sequence of immunotherapy with chemotherapy was taken into account, an RT dose of 57–63 Gy was associated with worse all-cause mortality compared to intermediate RT dose escalation among patients who started immunotherapy within ten weeks of starting chemotherapy (HR: 1.43, 95% CI: 1.06-1.92; p=0.02) (**Table 5**). There was no difference in all-cause mortality between RT dose of 57–63 Gy and IDE (64–74 Gy among patients who started immunotherapy between 10–14 weeks after starting chemotherapy (HR: 1.19, 95% CI: 0.98-1.44; p=0.08) or patients who started immunotherapy >14 weeks after starting chemotherapy (HR: 0.98, 95% CI: 0.78-1.24; p=0.88) (**Table 5**).

**Table 5:** Hazard ratio and its 95% CI of RT dose for all-cause mortality stratified by immunotherapy time after the start of chemotherapy.

Variable	Categories	HR 95% CI	P
Immunotherapy within 10 weeks of starting chemotherapy (42 days)			
RT dose	57–63 Gy	1.43 (1.06-1.92)	0.02
	64–74 Gy	Ref	
Immunotherapy between 11–14 weeks (71–98 days) after the start of chemotherapy			
RT dose	57–63 Gy	1.19 (0.98-1.44)	0.08
	64–74 Gy	Ref	
Immunotherapy >14 weeks (98 days) after the start of chemotherapy			
RT dose	57–63 Gy	0.98 (0.78-1.24)	0.88
	64–74 Gy	Ref	

The multivariable analysis was adjusted for age at diagnosis, gender, race, income, education, histology, comorbidity score, and treatment facility type.

## Discussion

Our current study is the most comprehensive retrospective study to investigate the role of IDE in the OS of unresectable stage III NSCLC patients who received definitive chemoradiation therapy in the era before and, most importantly, after the approval of immunotherapy. In this study, we focused on the comparisons between the standard dose of RT of 60Gy ± 10% vs. IDE (64-74Gy), as RT dose >74Gy have been abandoned after the RTOG 0617 results were reported. Indeed, data from NCDB show that only 160 (0.6%) of patients received dose higher than 74 Gy after 2015. We found that intermediate RT dose escalation 64–74 Gy is associated with improved OS compared to RT dose 57–63 Gy in the cohort before the era of immunotherapy for patients who received dcCRT. However, the improvement, though statistically significant (19.7 *vs.* 21.2 months), may not be clinically significant with less than 1.5 months difference. This result further justifies using 60Gy as the standard dose of definitive RT for stage III NSCLC for patients treated prior to the immunotherapy era.

We are mostly interested in the results from our study demonstrating that, in the era of immunotherapy, the survival benefit of intermediate RT dose escalation was only restricted to patients who started immunotherapy within six weeks of RT completion. There was no difference in all-cause mortality between RT dose 57–63 Gy and intermediate RT dose escalation of 64–74 Gy if immunotherapy was started between 7–10 weeks or >10 weeks after RT completion. A longer interval between the end of RT and the start of immunotherapy is associated with better OS, which seems to eliminate the need for dose escalation. In addition, we observed similar results for dose escalation when looking into the different intervals of starting immunotherapy after starting chemotherapy. Because NCDB does not record the ending date of chemotherapy, we decided to use the time interval of starting chemotherapy (with the assumption that the majority of the cases having chemotherapy started concurrently with RT; thus, 10 weeks after starting chemotherapy would be roughly translated to one month after completing chemotherapy) to the start of immunotherapy as another surrogate for sequencing of treatment. We observed the same pattern of the benefit of IDE only when immunotherapy was started in shorter intervals from the start of chemotherapy or, rather, from the end of chemoradiation therapy.

To better interpret the results and discussing on possible explanations, we would like to focus on a few major clinical factors that may render treating physicians to determine the timing of starting immunotherapy after dcCRT which, based on our clinical experience, would most likely rely on patients’ disease extensiveness at diagnosis and response to dcCRT, rapid *vs.* slow recovery or performance status after dcCRT due to toxicities from dcCRT, and/or accessibility to care. The last factor obviously is not consistent with our results.

One possibility to explain our findings is that among the patients who started immunotherapy within six weeks of RT completion, those who received an intermediate RT dose escalation were in better clinical conditions, for example, performance status to be candidates for such a high dose compared to those who received 57–63 Gy soon after dcCRT. However, it is less likely to be the case as the proportion of patients with a comorbidity score of zero, one, and two was similar between these two groups (**Table 1A**). We acknowledge the difference between comorbidity score and performance status but were obligated to study with estimation due to incomplete data for performance status in NCDB. It is also possible that these patients who started immunotherapy within 6 weeks had less severe disease to begin with and/or recovered more quickly from dcCRT. However, we observed an opposite trend in our data, *i.e.* higher proportion of more advanced stage such as stage IIIC patients started immunotherapy within 6 weeks after RT than later (**Table 1B**) while those patients were more likely to receive lower dose (**Table 1A**). This is quite likely per physicians’ preference with SD of RT concerning RT toxicities for more advanced disease/larger RT field size but starting immunotherapy in a shorter interval concerning tumor progression for these with advanced stage cancer despite of patients’ recovery from dcCRT toxicities, which could have caused more detrimental effects. Our data also suggest that IDE did not improve OS but may even have made OS worse for patients with more advanced stage of NSCLC by MVA (**Table 3B**). We hypothesize that the benefit of OS for IDE of RT for those started immunotherapy within six weeks after RT is contributed by the better outcomes in lower stage patients receiving IDE comparing with those of higher stage of cancer receiving SD, a group of patients more represented in the cohort of < 6 weeks. Nevertheless, this explanation is not against the idea of waiting for longer time thus SD RT (lower dose) can provide equivalent or better outcome than doing IDE at shorter time thus with potential less toxicities.

The second possible explanation of these findings is that starting immunotherapy early or too soon after completing dcCRT may undercount those patients who develop early disease progression during or soon after dcCRT. IDE could have reduced these early failures, thus providing an OS benefit. We do observe these rapid failure cases in the clinic as well as from the reports of PACIFIC trial, although quite rarely within the first one to two months after RT completion, as indicated by the PFS curves reported by Patel et al., 2020 (30). It is particularly uncommon these days with restaging imaging such as CT scans of the body becoming routine practice before starting immunotherapy. It is also interesting to point out that further analysis demonstrates that the survival benefits of IDE are limited to patients who started immunotherapy within 2 weeks of completion of RT (HR: 2.19, 95% CI: 1.32-3.62, p=0.002 standard dose vs. IDE) with a 2-year survival rate of 65% (95% CI: 57%-73%) and 84% (95% CI: 76%-92%) for standard dose and IDE, respectively. This finding also suggests against the second hypothesis with such a small-time window of uncounted potential disease failure generating >5% median overall survival difference.

The third hypothesis to explain our findings is that dcCRT may significantly affect the immune system, which requires time to recover before immunotherapy can take full effect, possibly related to eliminated infiltrating lymphocytes, a vital element of the immune response from conventional fractionated RT, and/or recruitment of myeloid-derived suppressor cells and tumor-associated macrophages both of which promote angiogenesis and tumor regrowth (31, 32). In this hypothesis, we assume that the first few cycles of immunotherapy, if started too early, *i.e.*, within the first 2 weeks or up to 6 weeks after completion of RT, may not provide any benefit, thus compromising the survival benefit of immunotherapy as supported by some clinical observations suggesting the benefit of starting immunotherapy not too soon after conventionally fractionated RT (33–36). Only in this situation may the benefit of IDE be revealed, which would have been masked by the full effects of immunotherapy if started with a sufficient delay. This hypothesis fits our results the best with better OS observed when starting immunotherapy later with at least six weeks of interval from completion of RT and/or chemotherapy in either standard or intermediate escalated dose of RT, and diminished OS benefit of IDE of RT when starting immunotherapy later than sooner. This hypothesis, if true, suggests that although IDE followed by immunotherapy within six weeks of RT completion was associated with improved OS compared to 57–63 Gy, dose escalation is not necessary when chemoradiation is followed by immunotherapy with a longer interval when the immune system in the patient recovers fully. A further subcategory comparison showed that among the patients who received the standard dose (57–63 Gy), starting immunotherapy > six weeks after RT completion had improved OS compared to ≤ six weeks (HR: 0.83, 95% CI: 0.72-0.95; p=0.01), while among patients who received IDE (64–74 Gy), there was no difference in the OS of patients who started immunotherapy ≤six weeks after RT completion and those who started immunotherapy > six weeks after RT completion (HR: 0.98, 95% CI: 0.79-1.23; p=0.88). These subcategory comparisons and the median OS reported in the result section further support that starting immunotherapy sooner is detrimental if the same dose level of standard RT is used. IDE may compensate for the detrimental effects of starting immunotherapy too early, i.e., < 6 weeks after RT.

The third hypothesis is consistent with the detrimental immune theory proposed by Jin et al., 2021 with a secondary analysis of the RTOG0617 trial reported that a higher dose of RT, especially to circulating immune cells, was correlated with decreased OS and indicated that a higher RT dose has a robust effect on immune cells depletion again suggesting that immunotherapy immediately after RT may not be the appropriate choice (37).

It is worthy noting that our data showing a trend of better OS as observed when immunotherapy was started later than early in both the standard RT dose and IDE cohorts, respectively, are contradicting the trends seen in PACIFIC and PACIFIC-R studies with both found improved survival associating with shorter interval although not statistically significant in either study with *post-hoc* analyses. In PACIFIC trial, survival time (OS or PFS) was counted from randomization which occurred after completion of dcCRT. Similarly, PACIFIC-R study defined that time-to-event endpoints were measured from the date that durvalumab was initiated, *i.e*. the PACIFIC-R index date. Neither study counted the survival time from the cancer diagnosis, unlike what we did in this study. A potential “lead-time” bias was introduced in both PACIFIC and PACIFIC-R study design with patients starting durvalumab earlier possibly being counted for “longer” PFS and OS simply due to counting the starting point earlier by likely a few months but not truly due to the longer survival time if counted from the diagnosis time. This discrepancy again strongly suggests the importance of future prospective randomized clinical trial(s) to make a definitive conclusion.

All these data suggest starting immunotherapy at the right timing post-dcCRT may improve OS. But it is unclear what is/are the most predictive and accessible clinical factor(s) to define the best timing of starting of immunotherapy. Many studies suggest that within tumor microenvironment (TME), it is not the total immune cells particularly T cells but rather the ratio of infiltrating immune cell such as CD8/CD4 cells that may indicate immunostimulating features of the tumor. Due to the challenges accessing tumor tissues, questions are raised but remain unclear whether systemic immune cell numbers/subtypes after dcCRT can be a surrogate of the cells in the tumor microenvironment although there are evidences suggesting so (38). Systemic immune inflammatory index (SII), which is commonly defined as a composite index integrating platelet counts, neutrophil and lymphocyte in peripheral blood, has been demonstrated as a prognostic factor in advanced stages of NSCLC (39, 40). How to correlate SII in individual patient with TME immune cell profiling and choose the right timing for delivering immunotherapy to maximize the tumor control remain unanswered which may require in future clinical trials more frequent sampling of immune cells in both peripheral blood and tumor tissue.

The strength of our study is the large sample size, which allowed us to adjust for some important factors and stratify by timing between RT completion and the initiation of immunotherapy so that the dose escalation question can be investigated in a detailed fashion with a large sample size. Limitations of this study include the study’s retrospective nature, lack of information about local control, toxicity, type of chemotherapy used concurrently with radiation, the type of immunotherapy, and if a single or multiple immunotherapies were recommended. We can only assume that at least majority of the cohort in our study received durvalumab per NCCN guideline because only durvalumab has been approved by FDA for unresectable stage III NSCLC as adjuvant immunotherapy after dcCRT per the phase III PACIFIC trial demonstrating significantly improved PFS and OS comparing with dcCRT only. Nevertheless, lack of information about the type of chemotherapy and RT dosimetric parameters or techniques such as 3D *vs*. IMRT planning are other limitations of our study. It is also noteworthy that we do not know whether patients had longer interval between the end of dcCRT to the start of immunotherapy is due to a better or worse treatment response to dcCRT. In clinical practice, physicians tend to initiate immunotherapy within 42 days per the PACIFIC trial design no matter how the tumor response is after dcCRT. For example, a complete clinical response of the tumor to dcCRT does not necessarily dictate a sooner or later initiation of durvalumab infusion. The interval in clinic is often dictated by how soon the follow-up CT body scans can be arranged after dcCRT, rather than by the treatment response. This question is very important but can only be answered with a prospective randomized trial stratifying patients with different responses.

## Conclusion

In this comprehensive analysis of the NCDB, RT dose escalation to 64–74 Gy was not associated with improved outcomes for patients diagnosed with stage III unresectable NSCLC who received immunotherapy > six weeks after chemoradiation. A longer interval between the end of RT and the start of immunotherapy improves OS, which may eliminate the need for dose escalation, with benefits only seen in patients starting immunotherapy within 6 weeks of RT completion. Our data support that the current standard dose of RT remains 60 Gy in 30 fractions even within the immunotherapy era, but raise the question whether there could be a benefit of a later onset of immunotherapy, *i.e.*, >6 weeks after completion of dcCRT. The findings warrant future prospective studies of combining immunotherapy with chemoradiation in patients who received intermediate RT dose escalation.

## Data Availability

The data analyzed in this study is subject to the following licenses/restrictions: The NCDB is not publicly available data, therefore, the data could not be shared. Requests to access these datasets should be directed to samin@unmc.edu.
